# 2-(4-Chloro­phen­yl)-5-fluoro-3-methyl­sulfinyl-1-benzofuran

**DOI:** 10.1107/S1600536810001157

**Published:** 2010-01-16

**Authors:** Hong Dae Choi, Pil Ja Seo, Byeng Wha Son, Uk Lee

**Affiliations:** a Department of Chemistry, Dongeui University, San 24 Kaya-dong Busanjin-gu, Busan 614-714, Republic of Korea; b Department of Chemistry, Pukyong National University, 599-1 Daeyeon 3-dong, Nam-gu, Busan 608-737, Republic of Korea

## Abstract

In the title compound, C_15_H_10_ClFO_2_S, the O atom and the methyl group of the methyl­sulfinyl substituent are located on opposite sides of the plane through the benzofuran fragment. The 4-chloro­phenyl ring is rotated out of the benzofuran plane, as indicated by the dihedral angle of 21.04 (4)°. The crystal structure exhibits a weak inter­molecular C—H⋯O hydrogen bond and a Cl⋯O halogen bond [Cl⋯O = 3.254 (1) Å].

## Related literature

For the crystal structures of similar 5-fluoro-2-(4-halophen­yl)-3-methyl­sulfinyl-1-benzofuran derivatives, see: Choi *et al.* (2009**a* ▶,*b* ▶,c* ▶). For the biological properties of benzofuran compounds, see: Aslam *et al.* (2006 ▶); Galal *et al.* (2009 ▶); Howlett *et al.* (1999 ▶). For natural products with benzofuran rings, see: Akgul & Anil (2003 ▶); Soekamto *et al.* (2003 ▶). For a review of halogen bonding, see: Politzer *et al.* (2007 ▶).
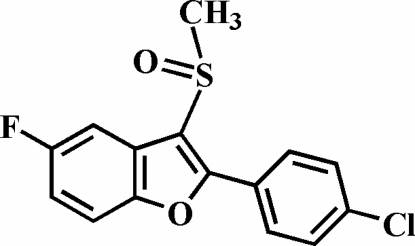


## Experimental

### 

#### Crystal data

C_15_H_10_ClFO_2_S
*M* *_r_* = 308.74
Triclinic, 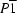

*a* = 7.7088 (5) Å
*b* = 8.5241 (5) Å
*c* = 11.6369 (7) Å
α = 74.572 (3)°
β = 70.893 (3)°
γ = 67.109 (3)°
*V* = 657.13 (7) Å^3^
*Z* = 2
Mo *K*α radiation
μ = 0.46 mm^−1^
*T* = 100 K
0.25 × 0.22 × 0.13 mm

#### Data collection

Bruker SMART APEXII CCD diffractometer
Absorption correction: multi-scan (*SADABS*; Bruker, 2009 ▶) *T*_min_ = 0.895, *T*_max_ = 0.942
10607 measured reflections
3020 independent reflections
2629 reflections with *I* > 2σ(*I*)
*R*_int_ = 0.032

#### Refinement

*R*[*F* ^2^ > 2σ(*F* ^2^)] = 0.032
*wR*(*F* ^2^) = 0.088
*S* = 1.03
3020 reflections
182 parameters
H-atom parameters constrained
Δρ_max_ = 0.42 e Å^−3^
Δρ_min_ = −0.27 e Å^−3^

### 

Data collection: *APEX2* (Bruker, 2009 ▶); cell refinement: *SAINT* (Bruker, 2009 ▶); data reduction: *SAINT*; program(s) used to solve structure: *SHELXS97* (Sheldrick, 2008 ▶); program(s) used to refine structure: *SHELXL97* (Sheldrick, 2008 ▶); molecular graphics: *ORTEP-3* (Farrugia, 1997 ▶) and *DIAMOND* (Brandenburg, 1998 ▶); software used to prepare material for publication: *SHELXL97*.

## Supplementary Material

Crystal structure: contains datablocks global, I. DOI: 10.1107/S1600536810001157/vm2017sup1.cif

Structure factors: contains datablocks I. DOI: 10.1107/S1600536810001157/vm2017Isup2.hkl

Additional supplementary materials:  crystallographic information; 3D view; checkCIF report

## Figures and Tables

**Table 1:** Hydrogen-bond geometry (Å, °)

*D*—H⋯*A*	*D*—H	H⋯*A*	*D*⋯*A*	*D*—H⋯*A*
C10—H10⋯O2^i^	0.93	2.52	3.347 (2)	148

Symmetry code: (i) 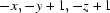.

**Fig. 1.**
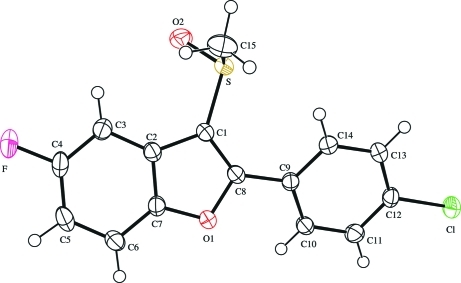
The molecular structure of the title compound with the atom numbering scheme. Displacement ellipsoids are drawn at the 50% probability level. H atoms are presented as a small spheres of arbitrary radius.

**Fig. 2.**
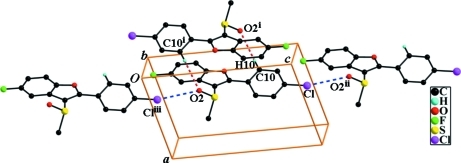
C–H···O and C–Cl···O interactions (dotted lines) in the crystal structure of the title compound. [Symmetry codes: (i) - x, - y + 1, - z + 1; (ii) x, y, z + 1; (iii) x, y, z - 1.]

## supplementary crystallographic information

### Comment

Benzofuran ring systems have drawn considerable interest in view of their biological properties (Aslam *et al.*, 2006; Galal *et al.*, 2009; Howlett *et al.* 1999) and their occurrence as natural products (Akgul & Anil, 2003; Soekamto *et al.*, 2003). As a part of our ongoing studies of the effect of side chain substituents on the solid state structures of 5-fluoro-2-(4-halophenyl)-3-methylsulfinyl-1-benzofuran analogues (Choi *et al.*, 2009*a,b,c*), we report the crystal structure of the title compound (Fig. 1).

The benzofuran unit is essentially planar, with a mean deviation of 0.006 (1) Å from the least-squares plane defined by the nine constituent atoms. The dihedral angle formed by the plane of the benzofuran and the 4-chlorophenyl ring is 21.04 (4)°. The crystal packing (Fig. 2) is stabilized by a weak intermolecular C–H···O hydrogen bond between the 4-chlorophenyl H atom and the oxygen of the S═O unit (Table 1). The molecular packing (Fig. 2) is further stabilized by a Cl···O halogen bond between the chlorine and the oxygen of the S═O unit [Cl···O2^ii^ = 3.254 (1) Å; C–Cl···O2 = 163.42 (6)°] (Politzer *et al.*,  2007).

### Experimental

77% 3-Chloroperoxybenzoic acid (291 mg, 1.3 mmol) was added in small portions to a stirred solution of 2-(4-chlorophenyl)-5-fluoro-3-methylsulfanyl-1-benzofuran (351 mg, 1.2 mmol) in dichloromethane (30 mL) at 273 K. After being stirred at room temperature for 3h, the mixture was washed with saturated sodium bicarbonate solution and the organic layer was separated, dried over magnesium sulfate, filtered and concentrated in vacuum. The residue was purified by column chromatography (hexane-ethyl acetate, 2:1 v/v) to afford the title compound as a colorless solid [yield 81 %, m.p. 442-443 K; *R*_f_ = 0.41 (hexane-ethyl acetate, 2:1 v/v)]. Single crystals suitable for X-ray diffraction were prepared by slow evaporation of a solution of the title compound in benzene at room temperature.

### Refinement

All H atoms were positioned geometrically and refined using a riding model, with C-H = 0.93 Å for aromatic H atoms and 0.96 Å for methyl H atoms, and with *U*_iso_(H) = 1.2*U*_eq_(C) for aromatic H atoms and 1.55*U*_eq_(C) for methyl H atoms.

### Crystal data

**Table:**

C_15_H_10_ClFO_2_S	*Z* = 2
*M**_r_* = 308.74	*F*(000) = 316
Triclinic, *P*1	*D*_x_ = 1.560 Mg m^−^^3^
Hall symbol: -P 1	Mo *K*α radiation, λ = 0.71073 Å
*a* = 7.7088 (5) Å	Cell parameters from 6597 reflections
*b* = 8.5241 (5) Å	θ = 2.6–27.6°
*c* = 11.6369 (7) Å	µ = 0.46 mm^−^^1^
α = 74.572 (3)°	*T* = 100 K
β = 70.893 (3)°	Block, colourless
γ = 67.109 (3)°	0.25 × 0.22 × 0.13 mm
*V* = 657.13 (7) Å^3^	

### Data collection

**Table:**

Bruker SMART APEXII CCD diffractometer	3020 independent reflections
Radiation source: Rotating Anode	2629 reflections with *I* > 2σ(*I*)
HELIOS	*R*_int_ = 0.032
Detector resolution: 10.0 pixels mm^-1^	θ_max_ = 27.6°, θ_min_ = 1.9°
φ and ω scans	*h* = −9→10
Absorption correction: multi-scan (*SADABS*; Bruker, 2009)	*k* = −11→11
*T*_min_ = 0.895, *T*_max_ = 0.942	*l* = −15→15
10607 measured reflections	

### Refinement

**Table:**

Refinement on *F*^2^	Primary atom site location: structure-invariant direct methods
Least-squares matrix: full	Secondary atom site location: difference Fourier map
*R*[*F*^2^ > 2σ(*F*^2^)] = 0.032	Hydrogen site location: difference Fourier map
*wR*(*F*^2^) = 0.088	H-atom parameters constrained
*S* = 1.02	*w* = 1/[σ^2^(*F*_o_^2^) + (0.0428*P*)^2^ + 0.2892*P*] where *P* = (*F*_o_^2^ + 2*F*_c_^2^)/3
3020 reflections	(Δ/σ)_max_ < 0.001
182 parameters	Δρ_max_ = 0.42 e Å^−^^3^
0 restraints	Δρ_min_ = −0.27 e Å^−^^3^

### Special details

**Table:**

Geometry. All esds (except the esd in the dihedral angle between two l.s. planes) are estimated using the full covariance matrix. The cell esds are taken into account individually in the estimation of esds in distances, angles and torsion angles; correlations between esds in cell parameters are only used when they are defined by crystal symmetry. An approximate (isotropic) treatment of cell esds is used for estimating esds involving l.s. planes.
Refinement. Refinement of F^2^ against ALL reflections. The weighted R-factor wR and goodness of fit S are based on F^2^, conventional R-factors R are based on F, with F set to zero for negative F^2^. The threshold expression of F^2^ > 2sigma(F^2^) is used only for calculating R-factors(gt) etc. and is not relevant to the choice of reflections for refinement. R-factors based on F^2^ are statistically about twice as large as those based on F, and R- factors based on ALL data will be even larger.

### Fractional atomic coordinates and isotropic or equivalent isotropic displacement parameters (Å^2^)

**Table:**

	*x*	*y*	*z*	*U*_iso_*/*U*_eq_	
Cl	0.33113 (7)	0.22955 (5)	1.02698 (4)	0.03627 (12)	
S	0.35910 (5)	0.20350 (5)	0.40485 (3)	0.02555 (11)	
F	0.16308 (18)	0.76845 (15)	0.01238 (10)	0.0474 (3)	
O1	0.17250 (15)	0.67346 (13)	0.48816 (10)	0.0258 (2)	
O2	0.25799 (18)	0.18174 (15)	0.32408 (12)	0.0356 (3)	
C1	0.2811 (2)	0.42581 (18)	0.41217 (13)	0.0232 (3)	
C2	0.2278 (2)	0.56740 (19)	0.31515 (14)	0.0243 (3)	
C3	0.2310 (2)	0.5825 (2)	0.19235 (14)	0.0299 (3)	
H3	0.2760	0.4867	0.1534	0.036*	
C4	0.1632 (3)	0.7478 (2)	0.13242 (15)	0.0325 (4)	
C5	0.0958 (3)	0.8951 (2)	0.18489 (16)	0.0339 (4)	
H5	0.0520	1.0032	0.1387	0.041*	
C6	0.0940 (2)	0.8808 (2)	0.30654 (15)	0.0302 (3)	
H6	0.0506	0.9771	0.3446	0.036*	
C7	0.1604 (2)	0.7151 (2)	0.36803 (13)	0.0248 (3)	
C8	0.2452 (2)	0.49619 (18)	0.51340 (13)	0.0231 (3)	
C9	0.2682 (2)	0.42787 (18)	0.63814 (13)	0.0225 (3)	
C10	0.1603 (2)	0.52936 (19)	0.73078 (14)	0.0251 (3)	
H10	0.0727	0.6383	0.7124	0.030*	
C12	0.3088 (2)	0.3053 (2)	0.87660 (13)	0.0258 (3)	
C11	0.1826 (2)	0.4693 (2)	0.84935 (14)	0.0277 (3)	
H11	0.1134	0.5386	0.9102	0.033*	
C13	0.4161 (2)	0.2011 (2)	0.78744 (14)	0.0277 (3)	
H13	0.4990	0.0903	0.8073	0.033*	
C14	0.3979 (2)	0.2645 (2)	0.66813 (14)	0.0269 (3)	
H14	0.4734	0.1971	0.6067	0.032*	
C15	0.6025 (2)	0.1816 (2)	0.31476 (18)	0.0396 (4)	
H15A	0.6681	0.0656	0.2979	0.059*	
H15B	0.6705	0.2072	0.3594	0.059*	
H15C	0.5994	0.2603	0.2385	0.059*	

### Atomic displacement parameters (Å^2^)

**Table:**

	*U*^11^	*U*^22^	*U*^33^	*U*^12^	*U*^13^	*U*^23^
Cl	0.0509 (3)	0.0319 (2)	0.0236 (2)	−0.00862 (18)	−0.01631 (17)	0.00016 (15)
S	0.0298 (2)	0.01997 (19)	0.0270 (2)	−0.00747 (15)	−0.00886 (15)	−0.00284 (14)
F	0.0699 (8)	0.0475 (7)	0.0278 (5)	−0.0194 (6)	−0.0255 (5)	0.0055 (4)
O1	0.0325 (6)	0.0201 (5)	0.0237 (5)	−0.0059 (4)	−0.0107 (4)	−0.0013 (4)
O2	0.0393 (6)	0.0307 (6)	0.0433 (7)	−0.0091 (5)	−0.0178 (5)	−0.0107 (5)
C1	0.0264 (7)	0.0195 (7)	0.0237 (7)	−0.0073 (6)	−0.0084 (5)	−0.0009 (5)
C2	0.0262 (7)	0.0227 (7)	0.0254 (7)	−0.0095 (6)	−0.0095 (6)	0.0003 (6)
C3	0.0373 (8)	0.0306 (8)	0.0252 (7)	−0.0135 (7)	−0.0111 (6)	−0.0025 (6)
C4	0.0391 (9)	0.0381 (9)	0.0227 (7)	−0.0154 (7)	−0.0151 (6)	0.0046 (6)
C5	0.0374 (9)	0.0282 (8)	0.0347 (9)	−0.0110 (7)	−0.0181 (7)	0.0087 (6)
C6	0.0335 (8)	0.0219 (7)	0.0340 (8)	−0.0065 (6)	−0.0135 (6)	−0.0006 (6)
C7	0.0267 (7)	0.0255 (7)	0.0234 (7)	−0.0093 (6)	−0.0100 (6)	−0.0003 (6)
C8	0.0230 (7)	0.0195 (7)	0.0256 (7)	−0.0058 (5)	−0.0080 (5)	−0.0013 (5)
C9	0.0245 (7)	0.0223 (7)	0.0224 (7)	−0.0092 (6)	−0.0081 (5)	−0.0013 (5)
C10	0.0261 (7)	0.0216 (7)	0.0258 (7)	−0.0060 (6)	−0.0079 (6)	−0.0019 (5)
C12	0.0301 (7)	0.0279 (8)	0.0211 (7)	−0.0114 (6)	−0.0099 (6)	0.0005 (6)
C11	0.0304 (8)	0.0274 (8)	0.0240 (7)	−0.0091 (6)	−0.0046 (6)	−0.0055 (6)
C13	0.0307 (8)	0.0229 (7)	0.0276 (8)	−0.0043 (6)	−0.0130 (6)	−0.0010 (6)
C14	0.0295 (8)	0.0248 (7)	0.0253 (7)	−0.0049 (6)	−0.0090 (6)	−0.0058 (6)
C15	0.0306 (8)	0.0324 (9)	0.0527 (11)	−0.0088 (7)	−0.0035 (8)	−0.0131 (8)

### Geometric parameters (Å, °)

**Table:**

Cl—O2^i^	3.254 (1)	C6—C7	1.382 (2)
Cl—C12	1.737 (2)	C6—H6	0.9300
S—O2	1.487 (1)	C8—C9	1.455 (2)
S—C1	1.768 (2)	C9—C14	1.396 (2)
S—C15	1.788 (2)	C9—C10	1.397 (2)
F—C4	1.361 (2)	C10—C11	1.381 (2)
O1—C7	1.373 (2)	C10—H10	0.9300
O1—C8	1.377 (2)	C12—C13	1.381 (2)
C1—C8	1.363 (2)	C12—C11	1.382 (2)
C1—C2	1.447 (2)	C11—H11	0.9300
C2—C7	1.388 (2)	C13—C14	1.381 (2)
C2—C3	1.393 (2)	C13—H13	0.9300
C3—C4	1.374 (2)	C14—H14	0.9300
C3—H3	0.9300	C15—H15A	0.9600
C4—C5	1.382 (3)	C15—H15B	0.9600
C5—C6	1.384 (2)	C15—H15C	0.9600
C5—H5	0.9300		
			
C12—Cl—O2^i^	163.42 (6)	C1—C8—C9	134.98 (14)
O2—S—C1	107.93 (7)	O1—C8—C9	114.44 (12)
O2—S—C15	105.99 (8)	C14—C9—C10	118.63 (13)
C1—S—C15	97.60 (8)	C14—C9—C8	122.01 (13)
C7—O1—C8	106.67 (11)	C10—C9—C8	119.35 (13)
C8—C1—C2	106.99 (13)	C11—C10—C9	120.57 (14)
C8—C1—S	126.32 (11)	C11—C10—H10	119.7
C2—C1—S	126.50 (11)	C9—C10—H10	119.7
C7—C2—C3	119.55 (14)	C13—C12—C11	121.54 (14)
C7—C2—C1	105.05 (13)	C13—C12—Cl	119.65 (12)
C3—C2—C1	135.40 (14)	C11—C12—Cl	118.80 (12)
C4—C3—C2	115.73 (15)	C10—C11—C12	119.31 (14)
C4—C3—H3	122.1	C10—C11—H11	120.3
C2—C3—H3	122.1	C12—C11—H11	120.3
F—C4—C3	117.68 (15)	C12—C13—C14	118.74 (14)
F—C4—C5	117.49 (15)	C12—C13—H13	120.6
C3—C4—C5	124.84 (15)	C14—C13—H13	120.6
C4—C5—C6	119.63 (15)	C13—C14—C9	121.16 (14)
C4—C5—H5	120.2	C13—C14—H14	119.4
C6—C5—H5	120.2	C9—C14—H14	119.4
C7—C6—C5	116.06 (15)	S—C15—H15A	109.5
C7—C6—H6	122.0	S—C15—H15B	109.5
C5—C6—H6	122.0	H15A—C15—H15B	109.5
O1—C7—C6	125.09 (14)	S—C15—H15C	109.5
O1—C7—C2	110.71 (13)	H15A—C15—H15C	109.5
C6—C7—C2	124.19 (14)	H15B—C15—H15C	109.5
C1—C8—O1	110.57 (13)		
			
O2—S—C1—C8	141.66 (14)	C1—C2—C7—C6	180.00 (14)
C15—S—C1—C8	−108.75 (15)	C2—C1—C8—O1	−0.06 (17)
O2—S—C1—C2	−32.73 (15)	S—C1—C8—O1	−175.34 (10)
C15—S—C1—C2	76.86 (15)	C2—C1—C8—C9	−179.09 (16)
C8—C1—C2—C7	−0.66 (16)	S—C1—C8—C9	5.6 (3)
S—C1—C2—C7	174.61 (11)	C7—O1—C8—C1	0.77 (16)
C8—C1—C2—C3	179.28 (17)	C7—O1—C8—C9	−179.99 (12)
S—C1—C2—C3	−5.5 (3)	C1—C8—C9—C14	22.0 (3)
C7—C2—C3—C4	−0.5 (2)	O1—C8—C9—C14	−156.97 (14)
C1—C2—C3—C4	179.56 (16)	C1—C8—C9—C10	−159.18 (17)
C2—C3—C4—F	−179.79 (14)	O1—C8—C9—C10	21.82 (19)
C2—C3—C4—C5	0.5 (3)	C14—C9—C10—C11	0.3 (2)
F—C4—C5—C6	−179.70 (15)	C8—C9—C10—C11	−178.49 (13)
C3—C4—C5—C6	0.0 (3)	C9—C10—C11—C12	−1.9 (2)
C4—C5—C6—C7	−0.5 (2)	C13—C12—C11—C10	1.3 (2)
C8—O1—C7—C6	179.96 (14)	Cl—C12—C11—C10	−178.53 (12)
C8—O1—C7—C2	−1.21 (16)	C11—C12—C13—C14	0.9 (2)
C5—C6—C7—O1	179.14 (14)	Cl—C12—C13—C14	−179.24 (12)
C5—C6—C7—C2	0.5 (2)	C12—C13—C14—C9	−2.6 (2)
C3—C2—C7—O1	−178.79 (13)	C10—C9—C14—C13	1.9 (2)
C1—C2—C7—O1	1.16 (16)	C8—C9—C14—C13	−179.25 (14)
C3—C2—C7—C6	0.0 (2)		

Symmetry codes: (i) *x*, *y*, *z*+1.

### Hydrogen-bond geometry (Å, °)

**Table:**

*D*—H···*A*	*D*—H	H···*A*	*D*···*A*	*D*—H···*A*
C10—H10···O2^ii^	0.93	2.52	3.347 (2)	148

Symmetry codes: (ii) −*x*, −*y*+1, −*z*+1.

## References

[bb1] Akgul, Y. Y. & Anil, H. (2003). *Phytochemistry*, **63**, 939–943. 10.1016/s0031-9422(03)00357-1 12895543

[bb2] Aslam, S. N., Stevenson, P. C., Phythian, S. J., Veitch, N. C. & Hall, D. R. (2006). *Tetrahedron*, **62**, 4214–4226.

[bb3] Brandenburg, K. (1998). *DIAMOND* Crystal Impact GbR, Bonn, Germany.

[bb4] Bruker (2009). *SADABS* *APEX2* and *SAINT* Bruker AXS Inc., Madison, Wisconsin, USA.

[bb5] Choi, H. D., Seo, P. J., Son, B. W. & Lee, U. (2009*a*). *Acta Cryst.* E**65**, o2084. 10.1107/S1600536809030190 PMC2970042 21577502

[bb6] Choi, H. D., Seo, P. J., Son, B. W. & Lee, U. (2009*b*). *Acta Cryst.* E**65**, o2115. 10.1107/S1600536809030992 PMC2970055 21577530

[bb7] Choi, H. D., Seo, P. J., Son, B. W. & Lee, U. (2009*c*). *Acta Cryst.* E**65**, o2608. 10.1107/S1600536809039312 PMC2971451 21578225

[bb8] Farrugia, L. J. (1997). *J. Appl. Cryst.* **30**, 565.

[bb9] Galal, S. A., Abd El-All, A. S., Abdallah, M. M. & El-Diwani, H. I. (2009). *Bioorg. Med. Chem. Lett* **19**, 2420–2428. 10.1016/j.bmcl.2009.03.069 19345581

[bb10] Howlett, D. R., Perry, A. E., Godfrey, F., Swatton, J. E., Jennings, K. H., Spitzfaden, C., Wadsworth, H., Wood, S. J. & Markwell, R. E. (1999). *Biochem. J* **340**, 283–289. PMC1220247 10229684

[bb11] Politzer, P., Lane, P., Concha, M. C., Ma, Y. & Murray, J. S. (2007). *J. Mol. Model* **13**, 305–311. 10.1007/s00894-006-0154-7 17013631

[bb12] Sheldrick, G. M. (2008). *Acta Cryst.* A**64**, 112–122. 10.1107/S0108767307043930 18156677

[bb13] Soekamto, N. H., Achmad, S. A., Ghisalberti, E. L., Hakim, E. H. & Syah, Y. M. (2003). *Phytochemistry*, **64**, 831–834. 10.1016/j.phytochem.2003.08.009 14559276

